# Roles of Plant Glycine-Rich RNA-Binding Proteins in Development and Stress Responses

**DOI:** 10.3390/ijms22115849

**Published:** 2021-05-29

**Authors:** Liqun Ma, Ke Cheng, Jinyan Li, Zhiqi Deng, Chunjiao Zhang, Hongliang Zhu

**Affiliations:** 1 The College of Food Science and Nutritional Engineering, China Agricultural University, Beijing 100083, China; lqma@cau.edu.cn (L.M.); 13164649232@163.com (K.C.); SY20193061114@cau.edu.cn (J.L.); zhiqideng@cau.edu.cn (Z.D.); 2 Supervision, Inspection & Testing Center of Agricultural Products Quality, Ministry of Agriculture and Rural Affairs, Beijing 100083, China; cc15809181@163.com

**Keywords:** glycine-rich RNA-binding protein, plant growth and development, stress responses, RNA immunoprecipitation, RNA post-transcriptional regulation

## Abstract

In recent years, much progress has been made in elucidating the functional roles of plant glycine-rich RNA-binding proteins (GR-RBPs) during development and stress responses. Canonical GR-RBPs contain an RNA recognition motif (RRM) or a cold-shock domain (CSD) at the N-terminus and a glycine-rich domain at the C-terminus, which have been associated with several different RNA processes, such as alternative splicing, mRNA export and RNA editing. However, many aspects of GR-RBP function, the targeting of their RNAs, interacting proteins and the consequences of the RNA target process are not well understood. Here, we discuss recent findings in the field, newly defined roles for GR-RBPs and the actions of GR-RBPs on target RNA metabolism.

## 1. Introduction

An increasing occurrence of extreme weather events (heatwaves, drought, torrential rains), together with dire projections regarding climate change, makes the improvement of crop resilience to environmental and pathogen stress of paramount importance for feeding a growing global population [1]. To this end, a greater understanding of molecular mechanisms involving stress tolerance genes is essential for genetic improvement of crop species.

One protein family, which has been linked to stresses such as cold, wounding, UV radiation, salinity and pathogen infection, is the glycine-rich RNA-binding proteins (GR-RBPs), which is a sub-family within the larger glycine-rich protein (GRP) superfamily [2]. GR-RBPs are widely distributed in organisms ranging from prokaryotes to eukaryotes, and in the plant kingdom, they have been identified in *Arabidopsis thaliana*, tobacco (*Nicotiana tabacum*), rice (*Oryza sativa*), maize (*Zea mays*), sweet potato (*Ipomoea batatas*), *Camelina sativa* and others [3,4,5,6,7,8]. GR-RBPs are critical for RNA processing and metabolism. In the last two years, more and more evidence that GR-RBPs can affect growth and development of different plant species has also been published. Processes of crucial importance for growth and development, such as the regulation of transcription and post-transcriptional modifications, are known to require the assistance of RNA-binding proteins (RBPs), however, in which capacity is not yet clear. GR-RBPs contain a canonical RNA recognition motif (RRM) or a cold-shock domain (CSD) at the N-terminus and a glycine-rich region at the C-terminus, and are involved in several processes, including pre-mRNA splicing, nucleocytoplasmic transport of RNA and RNA editing. GR-RBPs shuttling between the nucleus and the cytoplasm have been shown to bind their target transcripts in the nucleus to influence their processing, and so to understand the biological roles of GRPs in plants, the key is to find their direct target RNA and RNA-binding sites [9]. Recently, new technologies such as RNA immunoprecipitation (RIP) and UV crosslinking immunoprecipitation (CLIP) combined with high-throughput sequencing have been used to find target RBP RNAs and RNA-binding sites [10].

In summary, the biological functions of GR-RBPs have been described in various species, with special focus on regulation of gene expression, and RBPs are known to be involved in RNA mechanisms and several defense pathways; despite this, little is known about their structure/function relationship. To our knowledge, this is the first compilation of available data regarding the participation of plant GR-RBPs in various biological functions, such as development and stress responses, using different key technologies.

## 2. The GR-RBP Family

**Figure 1:**
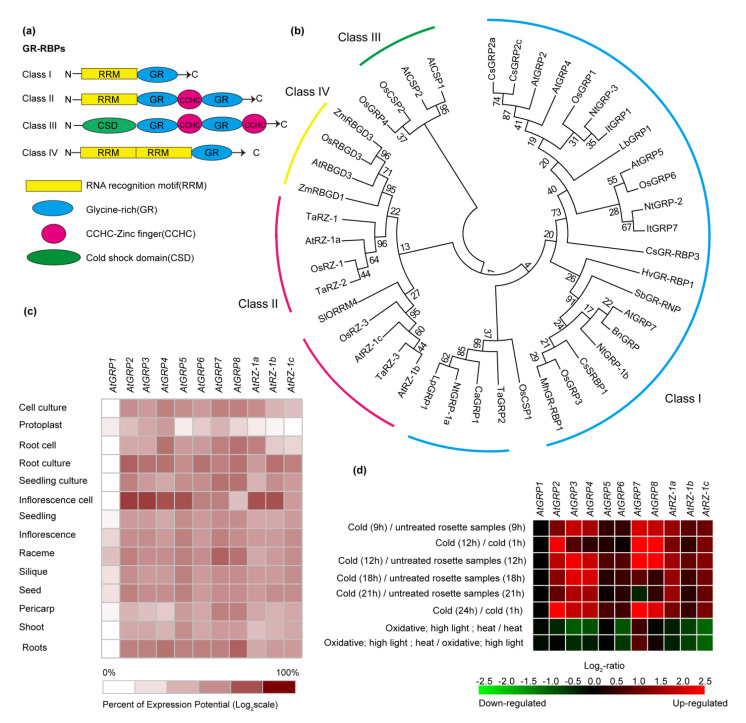
(**a**) Schematic representation of domain structures in plant glycine-rich RNA-binding proteins (GR-RBPs). Members of class I have an RNA recognition motif (RRM) at the N-terminus and a glycine-rich region at the C-terminus. Class II proteins, which are also called zinc finger-containing glycine-rich RNA-binding proteins (RZs), contain an RRM and a glycine-rich region separated by a CCHC-type zinc finger motif. Members of class III have an N-terminal cold shock domain (CSD) and a C-terminal glycine-rich region with two or more zinc finger motifs while class IV proteins have two RRMs and a glycine-rich region at the C-terminus. (**b**) Phylogenetic tree based on the alignment of protein sequences of GR-RBPs from sixteen plant species. These GR-RBPs are divided into four subclasses (denoted I–IV). Phylogenetic analyses were conducted in MEGA5. Species names are abbreviated as follows: Sl, *Solanum lycopersicum*; Bn, *Brassica napus*; Nt, *Nicotiana tabacum*; At, *Arabidopsis thaliana*; Cs, *Camelina sativa*; Ca, *Capsicum annuum*; Sb, *Sorghum bicolor*; Mh, *Malus hupehensis*; Hv, *Hordeum vulgare*; Cs, *Cucumis sativa*; Ca, *Capsicum annuum*; Os, *Oryza sativa*; Lp, *Lolium perenne*; Lb, *Limonium bicolor*; Ta, *Triticum aestivum*; It, *Ipomoea trifida*. (**c**) Expression profile of GR-RBPs in different tissues of *Arabidopsis thaliana*. (**d**) Expression profile of GR-RBPs from *Arabidopsis thaliana* under different treatments.

Plant glycine-rich proteins (GRPs) are characterized by a high content of glycine (Gly, 20–70%) and have been categorized into five classes, based on the presence of additional motifs and the arrangement of Gly repeats [11]. Among them, members of class IV, known as GR-RBPs, are particularly abundant in plants [12]. A canonical GR-RBP structure is shown in Figure 1a. This class is further divided into four subclasses (denoted I–IV). Subclass I contains one RNA recognition motif (RRM) domain as well as a glycine-rich (GR) motif, while subclass IV contains two RRM domains as well as the GR motif. The other two classes (II, III) both contain CCHC zinc-finger motifs, and are referred to as zinc finger-containing glycine-rich RNA-binding proteins (RZs). These have been found in *Arabidopsis thaliana* and rice (*Oryza sativa*) [5,11,13]. The biggest difference between these groups lies in the cold-shock domain (CSD), which is present only in subclass III. According to Table 1, the amount of subclass I in genomes of various plant species was much bigger than any other subclass (Figure 1b). Furthermore, we analyzed 11 GR-RBPs in different tissues and organs of *Arabidopsis thaliana*. As can be seen from Figure 1c, AtGRP1 was weakly expressed in all organs. However, the expression levels of the other genes were the highest in inflorescence cells and the lowest in protoplast.

## 3. Roles of GR-RBPs in Plant Growth and Development

Three distinct GR-RBP functions or development-associated expression patterns have been distinguished: stress-related seed germination, vegetative growth; and those related to flowering time and fruit ripening.

### 3.1. GR-RBPs in Stress-Related Seed Germination

Many GR-RBPs are known to play roles in stress-related seed germination (Table 2). The Arabidopsis genome encodes eight GR-RBPs in subclass I, and it includes three members which are involved in stress-related seed germination [14,15,16,17]. For example, AtGRP7 and AtGRP2 from *A. thaliana* have been shown to accelerate seed germination and seedling growth under low-temperature conditions [14,18]. In contrast, *AtGRP4*-overexpressing seeds were reported to have delayed germination during high-salt or dehydration stress [19]. Similarly, the rice genome encodes six GR-RBPs, in which only two members (OsGRP1 and OsGRP4) promoted seed germination and seedling growth under low temperatures [5,19]. The Arabidopsis AtRZ-1a, a zinc finger-containing glycine-rich RNA-binding protein, has a different function in the modulation of seed germination from AtRZ-1b and AtRZ-1c [20]. Overexpression of *AtRZ-1a* seeds were reported to have delayed germination, while the *AtRZ-1a* mutant resulted in accelerated seed germination under salt and freezing treatments [21]. In addition, AtRZ-1a suppressed seed germination through an abscisic acid (ABA)-dependent pathway [21]. By contrast, knocking out *AtRZ-1b* and *AtRZ-1c* expression suppressed the seed germination rate compared to WT [22]. Interestingly, among the three RZ family members present in wheat genomes, all three TaRZ-overexpressing transgenic Arabidopsis seeds showed delayed germination compared to WT under salt stress conditions. However, overexpression of *Triticum aestivum TaRZ-2* and *TaRZ-3* in *A. thaliana* resulted in retarded seed germination under dehydration stress conditions [23]. Furthermore, overexpression of *Malus prunifolia MpGR-RBP1* in *A. thaliana* resulted in accelerated seed germination under NaCl treatment [24]. Organelle RNA recognition motif-containing (ORRM) proteins are RNA editing factors in plants. In *A. thaliana*, ORRM3, ORRM4 and ORRM5 were previously characterized as GR-RBP3, GR-RBP5 and GR-RBP2 respectively, based on the presence of an RRM domain as well as the GR motif [25]. GR-RBP2 (ORRM5) was reported to accelerate seed germination under cold stress conditions, and Kwak et al. (2005) demonstrated that overexpressing *GR-RBP5* (ORRM4) in *A. thaliana* suppressed seed germination compared to WT plants under salt or dehydration stress conditions [19]. In summary, these studies demonstrate diverse functional roles for plant GR-RBPs in stress-related seed germination.

### 3.2. GR-RBP Function during Vegetative Growth

Studies have identified a role for RBPs in rice, where partial loss of RBP-P, a protein with two RRMs and a GR domain at the C-terminus, was reported to cause a broad range of phenotypes, including dwarfism, chlorophyll deficiency, sterility, late flowering and low spikelet fertility [51]. Similarly, a report by Tian et al. (2019) highlighted that the *RBP-L* knockout mutant also caused dwarfism, late flowering and smaller seeds in rice [52]. In addition, loss of function of *AtRZ-1b* and *AtRZ-1c* conferred defective phenotypes, including delayed seed germination, reduced stature and serrated leaves [39]. Notably, OsGRP1 was shown to promote cell expansion and elongation, and Wang et al. (2010) demonstrated that when overexpressing *OsGRP1* in the *A. thaliana* brassinosteroid-insensitive mutant, *bri1-5* partially suppressed its dwarf phenotype [40]. Furthermore, Staszak and Pawlowski (2014) suggested that a glycine-rich RNA-binding protein from Norway maple (*Acer platanoides*) may be directly involved in seed dormancy acquisition control [53].

### 3.3. GR-RBPs and Reproductive Growth

GR-RBPs have also been linked to the control of flowering time. In *A. thaliana*, partial loss of *AtGRP7* function resulted in late flowering and more rosette leaves when bolting compared to the wild-type control [30]. Additionally, in barley (*Hordeum vulgare*), HvGR-RBP1 participates in the control of the timing of anthesis and senescence and levels of grain protein [49]. Moreover, in wheat (*Triticum aestivum*), overexpression of *TaGRP2* resulted in late flowering compared with the wild type (WT) [48]. Furthermore, ORRM4 (GR-RBP5) and ORRM5 (GR-RBP2) are required for normal development, as *A. thaliana* loss-of-function mutants exhibit slower growth and delayed flowering time owing to defective mitochondrial editing [26,36,54], Finally, loss of tomato *SlORRM4* was reported to cause a delay in fruit ripening and defects in mitochondrial RNA editing, presumably as a consequence of significant changes in the editing of target RNAs [37].

## 4. GR-RBPs and Stress Responses

### 4.1. Temperature Treatments

GRP expression is known to be affected by different environmental stresses (e.g., cold, drought and salinity) in a number of plant species. Examples of studies describing their responses to altered temperatures are presented below. Notably, numbers of GR-RBPs responded sensitively to cold treatment in Arabidopsis leaves and roots. In subclass I, the level of *AtGRP2*, *AtGRP4* and *AtGRP7* transcripts increased significantly due to a decrease in the temperature. It can be seen from Figure 1d that in the cold treatment of multiple groups of experiments, the expression of *AtGRP2*, *AtGRP4* and *AtGRP7* was significantly upregulated. In addition, Kim et al. (2007) reported that overexpressing AtGRP2 enhanced freezing tolerance of Arabidopsis plants compared with the WT and *grp2*-knockout mutants [18]. Interestingly, heterologous expression of *AtGRP7* was seen to enhance growth in a cold-sensitive *Escherichia coli* mutant under low-temperature conditions [31]. Kim et al. (2010) observed that two members of the zinc finger-containing GR-RBPs, AtRZ-1a and AtRZ-1b, enhanced cold and freezing tolerance in *A. thaliana* [22]. However, AtRZ-1c appeared unable to complement cold sensitivity in the *E.coli* BX04 mutant [22]. Furthermore, overexpression of two cold-shock domain proteins (*AtCSDP1* and *AtCSDP2*) was shown to rescue cold-sensitive *AtGRP7* mutant plants from freezing damage [32]. Kim et al. (2010) reported that rice GRPs (OsGRP1, OsGRP4 and OsGRP6) contribute to the enhancement of cold and freezing tolerance, while three OsRZ proteins (OsRZ-1, OsRZ-2 and OsRZ-3) of OsRZ-2 have the ability to rescue *grp7*-knockout plants from cold conditions, but not OsRZ-1 and OsRZ-2 [13]. Furthermore, expression of *BnGRP1* from *Brassica napus* in *A. thaliana* was shown to result in accelerated seed germination and enhanced freezing tolerance grown under cold or freezing conditions [41]. Finally, the expression of three *CsGRP2* genes (*CsGRP2a*, *CsGRP2b* and *CsGRP2c*) from *Camelina sativa* L. was highly upregulated under cold stress and when heterologously expressed in *Escherichia coli* and had the ability to complement cold-sensitive mutants at low temperatures [8]. Other insights into roles of GR-RBPs in chilling responses came from studies of harvested cucumber (*Cucumis sativus*) fruit, where overexpressing *CsGR-RBP3* in *Arabidopsis* lines was shown to contribute to cold and freezing stress tolerance [42]. A role in cold acclimation has also been shown through studies of perennial ryegrass (*Lolium perenne*) in which *GRP1* transcription was significantly increased [43]. GR-RBPs are also thought to contribute to heat tolerance: *SbGRBP* from *Sorghum bicolor* expression is modulated by heat stress in seedlings [55] and the sweet potato genes *ItGRP1*, *ItGRP5* and *ItGRP7* are upregulated by heat stress [7]. The expression of *NtGRP-1a* and *NtGRP-3* in tobacco was strongly upregulated, while the expression of *NtRGP-1b* was unaffected by high-temperature treatment [4].

### 4.2. Salinity Stress

Salinity stress is one of the most significant factors limiting agricultural crop productivity [56] and improving crop salt tolerance is recognized as being essential for future sustainable food production. GR-RBPs have also been associated with salinity stress responses. As examples, overexpressing *Limonium bicolor LbGRP1* in tobacco improved its salt stress tolerance [44], and under 500 mM NaCl and 10 µm ABA treatments, the transcript levels of *S. bicolor SbGR-RNP* increased between four- and seven-fold [45]. A *Zoysia japonica* salt-induced glycine-rich RNA-binding protein, ZjGRP, resulted in increased salt sensitivity in *A. thaliana* when overexpressed [46]. Additionally, both AtGRP7 and AtGRP2 affect the growth and stress tolerance of *A. thaliana* plants under high salt and dehydration stress conditions [27,33], while the expression of AtGRP4 was downregulation by high salinity or dehydration stress. Finally, *MhGR-RBP1* from *Malus hupehensis* was reported to show a doubling of transcript levels in leaves 2 days after salt treatment [47]. Kwak et al. (2013) reported that the expression of *CsGRP2a* was highly upregulated, whereas the transcript levels of *CsGRP2b* and *CsGRP2c* were decreased under salt stress conditions [8].

### 4.3. Drought Stress

Drought stress is one of the major abiotic stresses having a direct impact on plant growth and limiting plant productivity [57,58]. Many scientific studies have focused on the development of drought-tolerant crop plants. As examples, overexpressing *AtGRP2* or *AtGRP7* in rice showed much higher recovery rates and grain yields compared with WT under drought stress conditions [28]. The expression of *NtGRP-1a* and *NtGRP-3* in tobacco was weakly upregulated under drought stress [4].

### 4.4. Biotic Stress

Plant virus-induced diseases of crops are of great importance to the agricultural industry due to the deleterious effects on product quality and yield [59]. Genetic resistance represents one approach to protect crops from viral infections [60] and this has been a motivating factor for studies of resistance genes.

Numerous studies have suggested that GR-RBP genes are likely involved in plant defenses. A GR-RBP from cucumber, CsRBP1, has been shown to inhibit the initial stages of *Zucchini yellow mosaic virus* (ZYMV) infection [29]. Another GRP, AtGRP7 from *A. thaliana*, has been shown to respond to challenge by *Pseudomonas syringae*, a necrotrophic bacterium (*Pectobacterium carotovorum* SCC1; formerly *Erwinia carotovora* SCC1), a necrotrophic fungus (*Botrytis cinerea*) and a biotrophic virus (*Tobacco mosaic virus*, TMV) [61]. In addition, a report by Yan et al. (2019) highlighted the importance of AtGRP7, AtGRP8 and AtGRP2 in inhibition of the early stages of ZYMV infection, and in particular the importance of the GR domain, which was shown to be both necessary and sufficient to block the early stages of infection [29].

An inverse correlation between expression of genes encoding GR-RBPs and virus resistance has also been reported. Huang et al. (2019) demonstrated that knockdown of GR-RBP *NgRBP* in *Nicotiana glutinosa* by virus-induced gene silencing enhanced *Potato virus X* (PVX) and *Cucumber mosaic virus* resistance [62]. In addition, a gene encoding GR-RBP2 was found to be highly expressed in a susceptible maize line associated with host hypersensitivity and susceptibility [63]. Finally, a study by Kim et al. (2015) supported silencing *CaGRP1* in pepper (*Capsicum annuum*) for enhanced resistance to *Xanthomonas campestris pv vesicatoria* (Xcv) infection [50]. Furthermore, CaGRP1 interacts with RECEPTOR-LIKE CYTOPLASMIC PROTEIN KINASE 1 (CaPIK1). These phosphorylated CaGRP1 and CaGRP1/CaPIK1 complexes suppress the expression of pathogen-related genes and negatively regulate CaPIK1-triggered cell death and defense responses.

## 5. Technologies Used to Investigate RNA–GR-RBP Interactions

The interaction between RNA-binding proteins and their target RNAs is a key factor in their fate and an important challenge in understanding the biological function of RNA-binding proteins is identifying the RNA targets in a cellular context. To date, the predominant methods for identifying unknown RNA targets have included protein immunoprecipitation, including RNA immunoprecipitation (RIP), individual nucleotide resolution crosslinking and immunoprecipitation (iCLIP), and high-resolution RIP-seq. Here, we describe three strategies for identifying RNA targets of GR-RBPs in plants.

### 5.1. RIP-qPCR

RIP is an antibody-based technique used to identify interactions between proteins and RNA targets in vivo. Specific RNA–protein complexes can be immunoprecipitated from a cellular lysate with an antibody raised against the protein of interest. RNA immunoprecipitation can be divided into two main classes: native and crosslinked.

**Figure 2:**
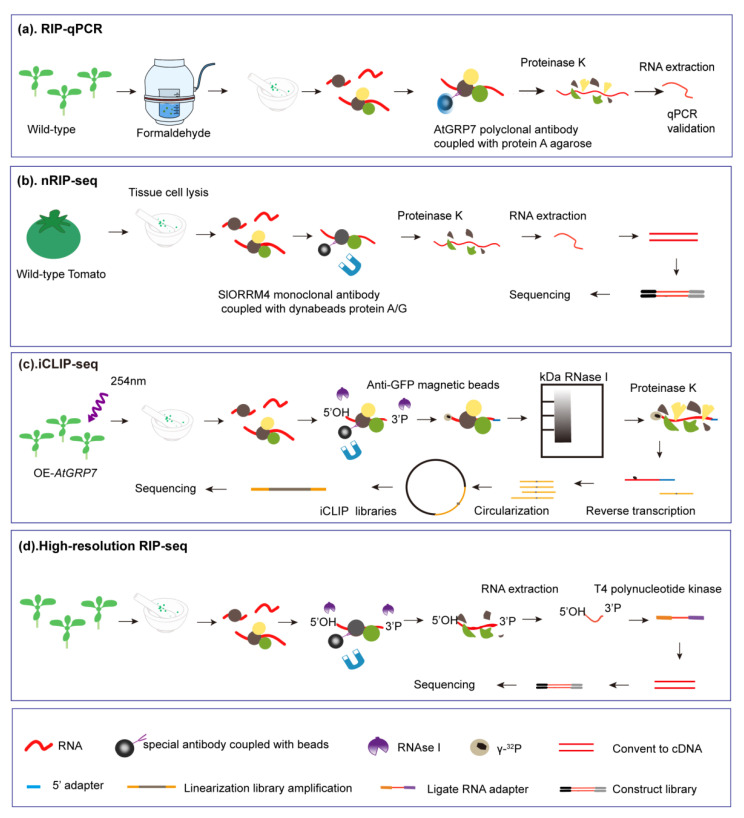
Workflow of RIP-qPCR, native RIP-seq, iCLIP and high-resolution RIP-seq analyses of plants. (**a**) RIP with a AtGRP7-specific antibody followed by qPCR to identify the binding status of AtGRP7 at the FLC locus. Point mutation of R^49^Q (Arg to Gln at the 49th amino acid) in the RRM motif was used as a negative control. Arabidopsis seedling tissue was cross-linked by 0.5% (*v*/*v*) formaldehyde. After tissue lysis, immunoprecipitation was performed using anti-AtGRP7 antibodies coupled with protein A agarose. Then, the protein was degraded by proteinase K, and RNA was extracted by acidic phenol/chloroform. The percentage of RIP-enriched RNA relative to that of input sample was determined by qRT-PCR. (**b**) For native RIP, tomato fruit (36 days post-anthesis (DPA)) cells were directly lysed and immunoprecipitation was performed using anti-SlORRM4 antibodies coupled with dynabeads protein A/G and proteins were digested using proteinase K. RNA extraction was performed and analyzed by next-generation sequencing. The negative control was IP from *slorrm4* mutant with anti-SlORRM4. (**c**) The GFP-tagged *AtGRP7* was expressed in the *grp7* mutant. Plant materials used for iCLIP were subjected to crosslinking with UV light 254 nm wavelength. After sample homogenization in liquid N_2_, a lysate was prepared and RNA–protein complexes were precipitated using specific antibodies coupled with magnetic beads. RNAs were fragmented by treatment with RNAse I and the fragments were radioactively labeled at the 5′ end only. After proteins were digested and RNAs were isolated, iCLIP sequencing libraries were prepared. In parallel, the negative control libraries were immunoprecipitated for GFP-only transgene plants and *AtGRP7 R4^9^Q-GFP* plants. (**d**) For high-resolution RIP-seq, tissues extracts were pre-treated with RNAse I. Immunoprecipitation was then performed using a specific antibody that recognized the protein of interest. After the proteins were digested, the RNA was phosphorylated using a T4 polynucleotide kinase and processed for sequencing using a Small RNA-seq Kit.

There are two methods for crosslinked RNA immunoprecipitation: irreversible UV crosslinking and reversible formaldehyde crosslinking [64]. Xiao et al. (2015) demonstrated that AtGRP7 binds to antisense *FLC* premRNA in vivo by RIP-qPCR using the crosslinkage of Arabidopsis seedling tissue (Figure 2a) [30]. Furthermore, Xiao et al. (2014) reported that TaGRP2 directly binds *TaVRN1* pre-mRNA with conserved binding sites through RIP [48].

Native RIP assays involve identification of RNA targets directly bound in the immunoprecipitated sample, while crosslinked RIP assays involve detection of direct and indirect binding sites on RNA targets (Figure 2b). RNA immunoprecipitation under native conditions without crosslinking combined with high-throughput sequencing (nRIP-seq) is a powerful technique that facilitates the direct target coding and non-coding RNAs associated with a particular protein [65]. Recently, nRIP-Seq analysis revealed that SlORRM4 is directly associated with 31 transcripts, 21 coding genes and 10 tRNAs, which were dramatically enriched in the immunoprecipitation of SlORRM4 (Figure 2b) [38]. The nRIP-Seq technique can find the RNAs targeted to GR-RBPs directly, but it cannot verify the interface between GR-RBPs and target RNAs.

### 5.2. ICLIP-Seq

The identification of RNA-binding sites facilitates the understanding of the molecular function of RNA-binding proteins. The iCLIP protocol starts with UV irradiation and then RNA–protein complexes are immunoprecipitated. After RNAse I digestion, the RNA 5′ ends are labeled with [γ-^32^P] ATP using a polynucleotide kinase and RNA–protein complexes are transferred to nitrocellulose membranes [9]. Finally, the associated RNAs are reverse transcribed into cDNAs, which are then PCR-amplified to generate libraries that are subjected to high-throughput sequencing (Figure 2c). In one study, the iCLIP approach revealed 858 transcripts, showing changes due to alternative splicing (AS) or polyadenylation in *atgrp7* knockout and *atgrp8* knockdown mutants, compared to *AtGRP7*-overexpressing plants [34]. *FATTY ACID DESATURASE 2* (FAD2), which has been shown to contribute to salt tolerance, was identified as a target RNA for AtGRP7 by iCLIP, and it was reported that overexpression of *AtGRP7* in *A. thaliana* had a negative effect on germination and seedling growth under salt stress conditions due to *FAD2* downregulation [34]. The disadvantage of this technique is that the irreversible UV crosslinking leads identification of RNA-binding sites to include both direct and indirect binding sites on RNA targets. In summary, these studies demonstrate that identification of the RNA target is critical for demonstration of the molecular regulatory mechanism underlying the effect of GR-RBPs on plant growth and development.

### 5.3. High-Resolution RIP-Seq

Recently, it was reported that modifying the standard RIP-seq protocol by adding a ribonuclease I digestion step after the chloroplast extraction step, but prior to antibody addition, led to the identification of an RNA-binding protein (HCF173) interaction with the target mRNA-binding site in *A. thaliana* (Figure 2d) [66]. In this case, the protein binding site was too short or had too low affinity to be identified with this method. However, this technology can be used to effectively explore the functional roles of GR-RBPs in gene regulation in the future.

## 6. Post-Transcriptional RNA Regulation by GR-RBPs

**Figure 3:**
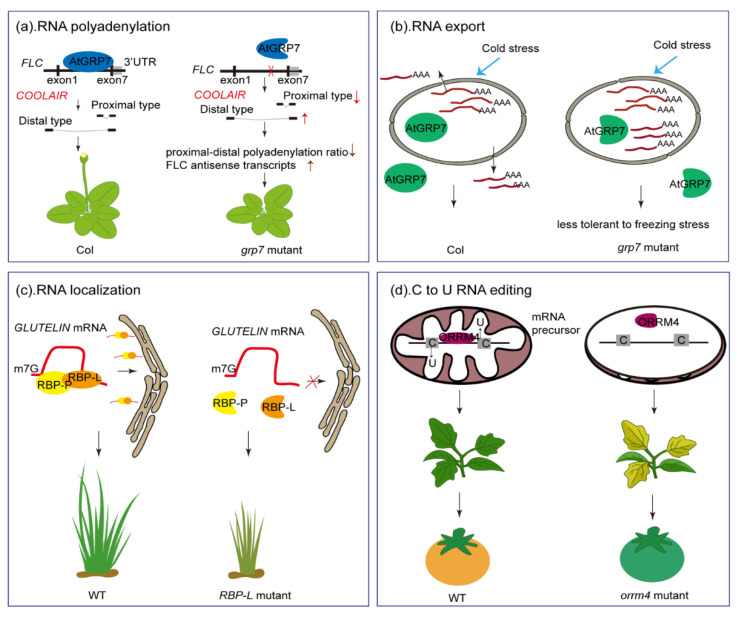
Cellular functions of diverse GR-RBPs involved in RNA metabolism during plant growth, development and stress responses. (**a**) AtGRP7 affects the polyadenylation site usage of *COOLAIR* transcripts, leading to an altered ratio of proximally–distally spliced variants. Loss of *AtGRP7* function leads to increased abundance of *FLC* antisense transcripts and a reduced proximal–distal polyadenylation ratio, resulting in late flowering compared with the wild type. (**b**) AtGRP7 is located in the nucleus and cytoplasm and is involved in mRNA export from the nucleus to the cytoplasm under cold stress conditions. In *grp7* mutants, the export of mRNA is impaired, leading to its accumulation in the nucleus, while mRNAs transcribed in the nucleus are efficiently exported to the cytoplasm in wild-type plants subjected to cold stress. (**c**) An insertional allele in rice, *RBP-L*, causes growth defects due to the loss of RBP and the consequent mislocalization of *GLUTELIN* target RNA. (**d**) Knocking out the mitochondrial RNA editing factor *ORRM4* in tomato causes defective mitochondrial editing, leading to yellowish seedlings and delayed fruit ripening.

RBPs are chaperones that bind RNA via one or multiple RRM domains, thereby changing the function or fate of the RNA targets. Here, we provide examples of the involvement of GR-RBPs in plant RNA metabolism, which collectively underscore the complexity of in vivo RNA-mediated processes (Figure 3).

### 6.1. GR-RBP Function in AS and Polyadenylation

AtGRP7 has been shown to affect AS and regulate pre-mRNA splicing in *A. thaliana* by binding to the target pre-mRNA and generating an intron-terminating alternatively spliced transcript, which is unstable and degraded through the nonsense-mediated decay pathway [35]. In a subsequent study, overexpression of *AtGRP7* was found to result in significant changes in the ratios of AS isoforms in 59 out of 288 analyzed AS events [67].

Cold-induced long antisense intragenic RNAs (*COOLAIR*) originate from the promoter adjacent to the *FLOWERING LOCUS C* (*FLC*) 3′UTR. Two structural variants of *COOLAIR* have been identified, terminating at proximal (sense intron 6) or distal (sense promoter) sites to repress *FLC* expression following cold exposure [68]. AtGRP7 has been shown to bind to *FLC* antisense pre-mRNA and the binding site is close to the polyadenylation site of the proximal *COOLAIR* structural type. Loss of *AtGRP7* function was observed to cause decreased levels of proximal type ASI (type I/total) and increased levels of distal type ASII (type II/total) *COOLAIR* to be expressed. The reduced proximal–distal polyadenylation ratio resulted in an increase in the total abundance of the functional sense *FLC* transcript and consequently influenced flowering time (Figure 3a) [30]. Wu et al. (2016) demonstrated that AtRZ-1c bound to *FLC*, promoting efficient splicing of *FLC* introns and repressing *FLC* transcription [39]

### 6.2. The Role of GR-RBPs in RNA Export

To experimentally validate mRNA subcellular localization, poly (A) in situ hybridization assays were performed in wild-type and *grp7* mutant plants. Strong fluorescence signals were observed in the nuclei of leaf cells in *grp7* plants subjected to cold stress while no noticeable fluorescence signals were present in the wild type [69], indicating thatAtGRP7 plays a role in mRNA export from the nucleus to the cytoplasm under cold stress conditions (Figure 3b) [69]. Through Northern blot analysis, Yan et al. (2019) showed that AtGRP7 binds ZYMV viral siRNA (vsiRNA), thus implicating it in cell-to-cell delivery of siRNA, leading to activation of the RNAi pathway in neighboring cells [29].

### 6.3. RNA Localization

RNA-binding proteins have been shown to play critical roles in transporting mRNA to specific subcellular locations for localized translation. Recent studies identified two RBPs, RBP-P and RBP-L, containing two and three RRM domains and a GR domain at the C-terminal, respectively [51,52], which specifically bound to the *glutelin* zipcode mRNA sequences, thereby regulating *glutelin* mRNA localization (Figure 3c) [51,52]. Glutelin RNAs are asymmetrically distributed on distinct endoplasmic reticulum (ER) subdomains of the cortical–ER complex [52].

### 6.4. The Role of GR-RBPs in C-to-U RNA Editing

RNA editing allows modification of genetic information at the transcriptional level by inserting, knocking out and replacing RNA base sequences, and C (cytidine) to U (uridine) RNA editing in plants occurs in chloroplast and mitochondrial transcripts [70]. It was reported that the knockout of ORRM4 (glycine-rich RNA-binding protein 5), which functions as a major mitochondrial editing factor, in *A. thaliana* caused defective mitochondrial editing in 44% of the mitochondrial sites surveyed [36]. In addition, knocking out *ORRM5* expression resulted in an increase of the editing extent in 14% of the mitochondrial sites [26]. The knockout *orrm5* mutant reduced the splicing efficiency of the first *nad5* intron, causing slower growth [26]. Furthermore, SlORRM4 from tomato was also shown to be a key RNA editing factor and responsible for 61% of the 552 C-to-U editing events in mitochondria. SlORRM4-mediated RNA editing events normally cause about 56% of the expressed mitochondrial genes to develop missense mutations, and defects in RNA editing in the *slorrm4* mutant had a significant effect on the expression levels of these mitochondrial genes [38]. Moreover, using nRIP-seq, 19 SlORRM4 RNA targets were identified, which were mostly subunits of mitochondrial respiratory chain complexes [38]. Defective RNA editing in the *slorrm4* mutant resulted in yellowish seedlings and a major delay in the initiation of tomato fruit ripening (Figure 3d) [38].

## 7. Conclusions and Discussion

GR-RBPs are involved in a range of abiotic and biotic stress responses, including cold adaptation and responses to pathogens. Czolpinska et al. (2018) demonstrated that GRPs play important roles in responses to diverse stress conditions [71]. In this review, we systematically summarized GR-RBPs that are involved in RNA metabolism during plant growth and development. Importantly, identification of the RNA target is critical for demonstration of the molecular regulatory mechanism underlying the function of GR-RBPs. The RRM domain confers RNA-binding (and potentially DNA-binding) activity, allowing the regulation of genes with multiple roles. Additional regulatory roles for GR-RBPs include the regulation of pre-mRNA alternative splicing, mRNA export and mRNA localization. Importantly, members of the GR-RBP family have been shown in mammals to be extensively involved in various forms of neurodegenerative diseases and neurodevelopmental disorders. Unlike in plants, it has been reported that in mammals, GRPs not only regulate the transcription of target genes but also affect the translation efficiency of proteins. For instance, expression of RNA-binding motif 3 (Rbm3), a cold-induced member of the GRP family, can increase total protein synthesis under both physiological and mild hypothermic temperatures in N_2_a cells [72]. RNA-binding motif single-stranded interacting protein 3 (Rbms3), a glycine-rich RNA-binding protein, can bind to the 3′ untranslated region (UTR) of *pancreas-associated transcription factor 1a* (*Ptf1a*) mRNA, regulating the accumulation of the encoded Ptf1a protein to maintain pancreas development [73]. Among the many important unanswered questions regarding the role of GR-RBPs in plants is whether they are associated with translational regulation of their RNA targets. Biomacromolecules contain between tens and several thousands of protein and RNA species which aggregate to form membraneless organelles [74]. These condensates often contain RBPs with large intrinsically disordered regions (IDRs) that can promote phase separation [75]. Liquid–liquid phase separation occurs in diverse biological processes, including signal transduction, gene expression regulation, higher-order chromatin organization, cell division, etc. [76]. The study of protein phase separation is an emerging field in animal and yeast research but is still not common in plants [77]. Xie et al. (2020) showed that nuclear dicing bodies (D-bodies, containing DICER-LIKE 1, HYPONASTIC LEAVES 1 and SERRATE) are phase-separated condensates and essential for efficient miRNA processing [78]. Furthermore, Huang et al. (2021) reported that reactive oxygen species (ROS) regulate reversible protein phase separation to direct stem cell fate for flowering transition. Proteins contain large IDRs that are enriched in amino acids, such as glycine, serine, glutamine, asparagine, phenylalanine and tyrosine [79]. Therefore, many GR-RBPs are predicted by the presence of IDR sequences to be involved in phase separation under specific physiological conditions. Functional characterization of newly identified GR-RBPs and their RNA targets will pave the way for a better understanding of post-transcriptional and translational regulation of plant growth and development.

## Figures and Tables

**Table 1:** Number of predicted genes for each GR-RBP subclass in various plants.

Plant	Subclass I	Subclass II	Subclass III	Subclass IV	Total
*Arabidopsis thaliana*	8	3	2	5	18
*Oryza sativa*	6	3	2	4	15
*Zea mays*	6	6	2	9	23
*Theobroma cacao*	6	0	0	9	15
*Brassica rapan* L. *ss* *P. pekinensis*	15	4	5	6	30
*Gossypium raimondii*	11	6	6	9	32
*Gossypium arboreum*	14	4	7	12	37

**Table 2:** Description of the identified plant glycine-rich RNA binding proteins.

Gene	Subclass	Gene Source	Growth Phenotype	Roles	References
*AtGRP2* *ORRM5*	I	*Arabidopsis thaliana*	Under cold stress accelerate seed germination and seedling growth, under high salt and dehydration stress conditions affect plants growth and stress tolerance, slow growth and late flowering	Overexpressing enhances freezing tolerance of Arabidopsis plants, *AtGRP2* in inhibition of the early stages of ZYMV infection, mitochondrial RNA editing	[14,25,26,27,28,29]
*AtGRP4*	I	*Arabidopsis thaliana*	Overexpressing seeds delayed germination during high salt or dehydration stress	The transcripts increase under cold stress, and downregulated by high salinity and dehydration stress	[18,19]
*AtGRP7*	I	*Arabidopsis thaliana*	Affect the growth and stress tolerance of *A. thaliana* plants, influence flowering time, overexpressing in rice shows higher recovery rates and grain yields	The transcripts increase significantly under cold stress, influence AS or polyadenylation, influence mRNA export from the nucleus to the cytoplasm under cold stress conditions,	[18,27,28,30,31,32,33,34,35]
				involvement in plant defenses, such as *Pseudomonas syringae* and *TMV*	
*ORRM4* *GR-RBP5*	I	*Arabidopsis thaliana*	Slow growth and late flowering	Mitochondrial RNA editing	[19,36]
*SlORRM4*	I	*Solanum lycopersicum*	Delayed tomato fruit ripening	Mitochondrial RNA editing	[37,38]
*AtRZ-1a*	III	*Arabidopsis thaliana*	Enhances tolerance to cold stress in *A. thaliana*	Overexpressing in salt and cold stress retards seed germination	[21,22]
*AtRZ-1b*	III	*Arabidopsis thaliana*	Enhances tolerance to cold stress, *AtRZ-1b* and *AtRZ-1c* knockout mutants delayed seed germination, reduced stature and serrated leaves	Promote efficient splicing of *FLC* introns and repress *FLC* transcription	[22,39]
*AtRZ-1c*	III	*Arabidopsis thaliana*	*AtRZ-1b* and *AtRZ-1c* knockout mutants delayed seed germination, reduced stature and serrated leaves	Promote efficient splicing of *FLC* introns and repress *FLC* transcription	[22,39]
*TaRZ* *-* *2*	III	*Triticum aestivum*	Overexpressing retards seed germination under dehydration stress condition	Not determined	[23]
*TaRZ* *-* *3*	III	*Triticum aestivum*	Overexpressing retards seed germination under dehydration stress condition	Not determined	[23]
*OsGRP1*	I	*Oryza sativa*	Overexpressing suppresses the dwarf phenotype of *Arabidopsis bri1-5* mutant, under low temperatures promotes seed germination and seedling growth	Promotes cell expansion and elongation, enhances freezing tolerance	[5,40]
*OsGRP4*	I	*Oryza sativa*	Under low temperatures promotes seed germination and seedling growth	Enhances tolerance to cold stress	[5]
*OsGRP6*	I	*Oryza sativa*	Not determined	Enhances tolerance to cold stress	[5]
*OsRZ-2*	III	*Oryza sativa*	Under low temperatures rescues *grp7*- knockout plants	Not determined	[13]
*NtGRP-1a*	I	*Nicotiana tabacum*	Not determined	Upregulation of abundance during heat and drought stress	[4]
*NtGRP-3*	I	*Nicotiana tabacum*	Not determined	Upregulation of abundance during heat and drought stress	[4]
*BnGRP1*	I	*Brassica napus*	Under cold stress accelerates seed germination	Enhances tolerance to cold stress	[41]
*CsGRP2*	I	*Camelina sativa*	Complement cold-sensitive mutants at low temperatures	Upregulation of cold stress	[8]
*CsGRP3*	I	*Cucumis sativ* *a*	Overexpressing contributes to cold and freezing stress tolerance	Not determined	[42]
*LpGRP1*	I	*Lolium perenne*	Not determined	Upregulation of cold stress	[43]
*ItGRP1* *ItGRP5* *ItGRP7*	I	*Ipomoea trifida*	Not determined	Upregulation of heat stress	[7]
*LbGRP1*	I	*Limonium bicolo* *r*	Not determined	Improves tolerance to salt stress	[44]
*SbGR-RNP*	I	*Sorghum bicolor*	Not determined	Upregulation of salt stress	[45]
*ZjGRP*	I	*Zoysia japonica*	Overexpressing increases salt sensitivity in *A. thaliana*	Not determined	[46]
*MhGR-RBP1*	I	*Malus hupehensis*	Not determined	Upregulation of salt stress	[47]
*CsSRBP1*	I	*Cucumis sativus*	Not determined	Inhibits the initial stages of *Zucchini yellow mosaic virus* (ZYMV) infection	[29]
*TaGRP2*	I	*Triticum aestivum*	Not determined	Inhibits transcript accumulation of *TaVRN1*	[48]
*HvGR-RBP1*	I	*Hordeum vulgare*	Not determined	Involvement in the timing of anthesis, senescence and levels of grain protein	[49]
*CaGRP1*	I	*Capsicum annuum*	Not determined	Resistance to *Xanthomonas campestris pv vesicatoria* (Xcv) infection	[50]
